# Potential application of measuring serum infliximab levels in rheumatoid arthritis management: A retrospective study based on KURAMA cohort data

**DOI:** 10.1371/journal.pone.0258601

**Published:** 2021-10-13

**Authors:** Kazuto Nakae, Sho Masui, Atsushi Yonezawa, Motomu Hashimoto, Ryu Watanabe, Koichi Murata, Kosaku Murakami, Masao Tanaka, Hiromu Ito, Kotoko Yokoyama, Noriko Iwamoto, Takashi Shimada, Miyuki Nakamura, Masaya Denda, Kotaro Itohara, Shunsaku Nakagawa, Yasuaki Ikemi, Satoshi Imai, Takayuki Nakagawa, Makoto Hayakari, Kazuo Matsubara

**Affiliations:** 1 Department of Clinical Pharmacology and Therapeutics, Kyoto University Hospital, Kyoto, Japan; 2 Graduate School of Pharmaceutical Sciences, Kyoto University, Kyoto, Japan; 3 Department of Advanced Medicine for Rheumatic Diseases, Graduate School of Medicine, Kyoto University, Kyoto, Japan; 4 Department of Rheumatology and Clinical Immunology, Graduate School of Medicine, Kyoto University, Kyoto, Japan; 5 Shimadzu Corporation, Kyoto, Japan; 6 Shimadzu Scientific Instruments, Bothell, Washington, United States of America; Nippon Medical School, JAPAN

## Abstract

Infliximab (IFX) therapy has considerably improved the treatment of rheumatoid arthritis (RA). However, some patients still do not respond adequately to IFX therapy, or the efficacy of the treatment diminishes over time. Although previous studies have reported a relationship between serum IFX levels and therapeutic efficacy, the potential applications of IFX therapeutic drug monitoring (TDM) in clinical practice remain unclear. The purpose of this study was to investigate the potential applications of IFX TDM by analyzing a Japanese cohort database. Data were collected retrospectively from the Kyoto University Rheumatoid Arthritis Management Alliance cohort between January 1, 2011, and December 31, 2018. Serum IFX levels were measured using a liquid chromatography-tandem mass spectrometer. Out of the 311 RA patients that used IFX, 41 were eligible for the analysis. Serum IFX levels were significantly higher in responders than in non-responders. An optimal cut-off value was determined to be 0.32 μg/mL based on a receiver operating characteristic curve. At the IFX measurement point, a better therapeutic response was observed in the high IFX group (n = 32) than in the low IFX group (n = 9). Conversely, at the maximum effect point, when DAS28-ESR was the lowest between IFX introduction and measurement points, there were no differences in responder proportions between the low and high IFX groups. IFX primary ineffectiveness could be avoided with appropriate dose escalation without blood concentration measurement in clinical practice. In conclusion, IFX TDM could facilitate the identification of secondary non-responders and in turn, proper IFX use.

## Introduction

Infliximab (IFX) is a chimeric monoclonal antibody composed of human constant and murine variable regions that specifically bind to tumor necrosis factor alpha (TNF-α). IFX therapy has substantially improved the treatment of rheumatoid arthritis (RA). The result of Anti-Tumor Necrosis Factor Trial in Rheumatoid Arthritis with Concomitant Therapy (ATTRACT) study has revealed that IFX therapy provided clinical benefits and halted joint damage progression [1, 2]. However, in some patients, the efficacy of IFX therapy is not adequate, or is gradually lost with the lapse of the treatment [3–6]. It has also been reported that secondary non-response occurs in approximately a half of RA patients during the first year of its treatment [7]. In addition, another study has shown that IFX discontinuation rate due to inefficacy was 32.1% at 36 months [8]. One of the current challenges in IFX therapy is to avoid secondary non-response in long-term treatment.

The pharmacokinetic mechanisms of therapeutic antibodies have largely been clarified. The development of anti-drug antibodies (ADAs) is associated with low serum drug levels and non-response [9–11]. Previous studies have shown that approximately 10–60% of RA patients receiving IFX developed ADAs against IFX within the first 6 months [12–15]. In addition to ADAs, baseline TNF-α level is another factor that reduces serum IFX levels [16]. Furthermore, FcRn (neonatal Fc receptor) function influences the pharmacokinetics of therapeutic antibodies [17, 18]. High inter- and intra-individual variabilities in monoclonal antibody pharmacokinetics have been reported [19]. Consequently, therapeutic strategies that take into account IFX pharmacokinetics variability should be developed.

Therapeutic drug monitoring (TDM) has facilitated the optimal and appropriate use of immunosuppressive drugs and antiepileptic drugs, etc. Based on the serum concentrations of drugs, dosages can be adjusted to appropriate therapeutic concentrations and ranges. Some studies have demonstrated that clinical responses to IFX therapy are associated with serum IFX levels. A prospective, randomized, double-blind study (the RISING study) has reported a significant correlation between serum IFX levels and disease activity score in 28 joints (DAS28)-remission [20]. A non-interventional retrospective study has also reported that high serum IFX levels are related to good responses at 52 weeks from baseline [15]. Although a relationship between serum IFX levels and its therapeutic benefits has been described in several studies [15, 20–22], it remains unclear how IFX TDM could be applied in clinical practice.

Here, we conducted a retrospective cohort study by enrolling consecutive RA patients treated with IFX in a cohort, and investigated the practicality of IFX TDM in clinical practice. Furthermore, we measured ADA levels to evaluate its correlation with serum IFX levels.

## Materials and methods

### Patients

The study subjects were enrolled from the Kyoto University Rheumatoid Arthritis Management Alliance (KURAMA) cohort, which was established in 2011 by the Center for Rheumatic Diseases at Kyoto University Hospital. The cohort aims to provide strict RA control and to use patient clinical and laboratory data in clinical investigations, as described previously [23]. All patients fulfilled the revised 1987 American College of Rheumatology (ACR) or the 2010 ACR/European League Against Rheumatism (EULAR) classification criteria for RA. Written informed consent to enroll in this retrospective cohort study was obtained from all the patients. The present study adhered to the principles of the Declaration of Helsinki, and was approved by the Medical Ethics Committee of Kyoto University Graduate School and Faculty of Medicine (R0357).

**Fig 1:**
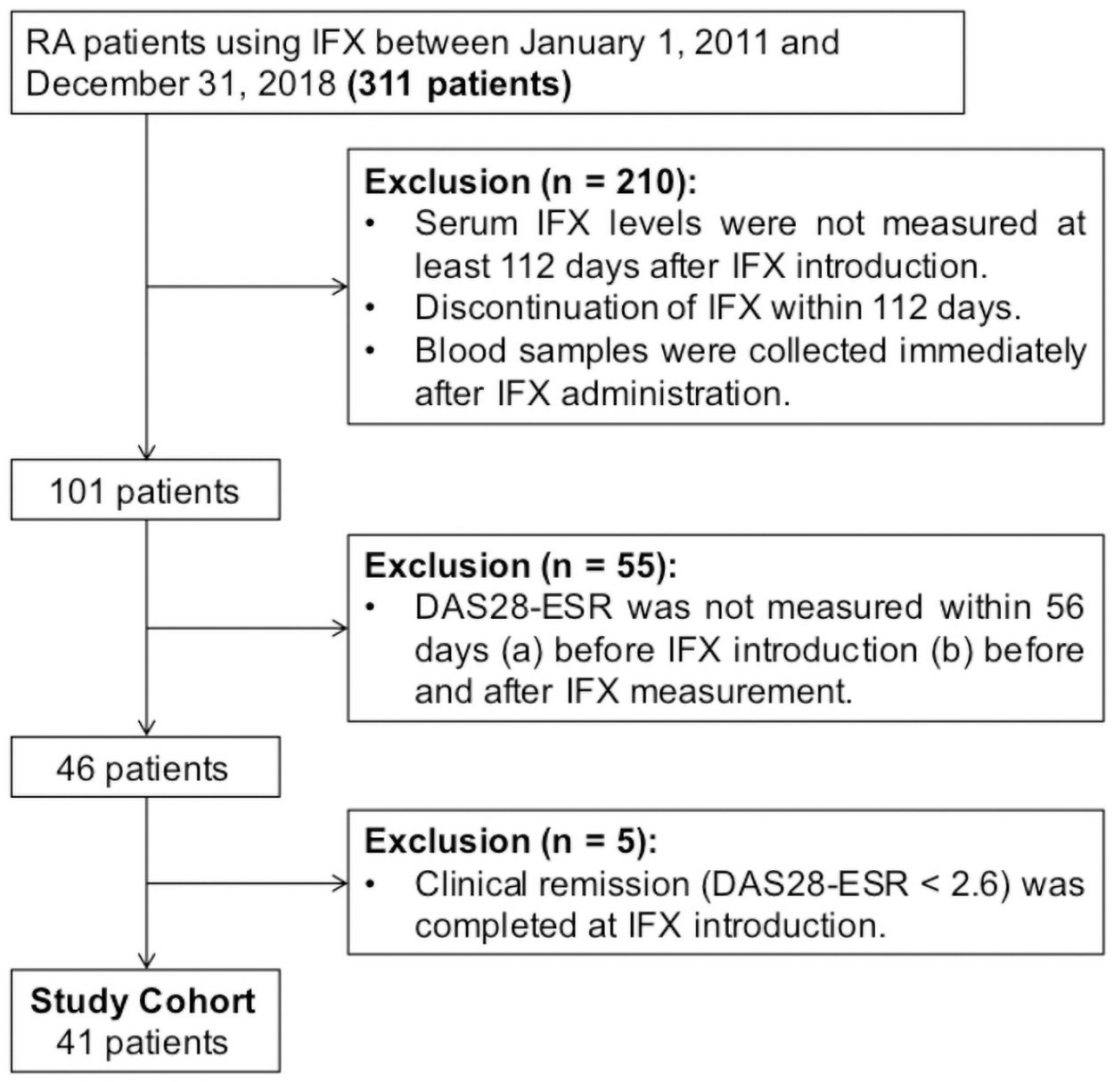
Flowchart on patient inclusion and exclusion. Abbreviations: IFX, infliximab; DAS28-ESR, the 28 joint disease activity score incorporating erythrocyte sedimentation rate; RA, rheumatoid arthritis.

KURAMA cohort data between January 1, 2011 and December 31, 2018 were used in the present study. IFX was administered at 0, 2, and 6 weeks in the induction phase and thereafter every 8 weeks in the maintenance phase. In this study, 112 days (16 weeks) after the initiation was defined as the initiation of the maintenance phase. Out of the 311 RA patients with IFX therapy, 210 were excluded, because their serum IFX levels were not obtained during maintenance therapy (at least 112 days after IFX introduction). In addition, 55 patients were excluded due to lack of the 28-joint disease activity score incorporating erythrocyte sedimentation rate (DAS28-ESR) data at initiation and measurement point of IFX. When DAS28-ESR was not recorded on the IFX initiation and measurement days, DAS28-ESR at the visit before IFX initiation and the visit before or after IFX measurement were allowed to be used. Five patients who had already completed clinical remission (DAS28-ESR <2.6) before IFX introduction were also excluded, and 41 patients were eligible for further analysis (Fig 1).

### Data collection and evaluation of disease activity

Clinical characteristics included age, body weight, sex, RA disease duration, IFX treatment duration, weekly methotrexate (MTX) dose, oral glucocorticoid use, conventional synthetic disease modifying anti-rheumatic-drug (csDMARD) use, tender joint count, swollen joint count, C-reactive protein (CRP) level, and rheumatoid factor (RF). Actarit, aurothiomalate, auranofin, bucillamine, iguratimod, leflunomide, mizoribine, salazosulfapyridin, cyclosporine, and tacrolimus were considered as csDMARDs. RA disease activity was evaluated based on clinical disease activity index (CDAI), simplified disease activity index (SDAI), physical disability by health assessment questionnaire-disability index (HAQ-DI), and DAS28-ESR. Baseline was defined as the last data within 3 months before IFX introduction. Patients achieving good or moderate responses to IFX therapy according to the EULAR response criteria were defined as “responders,” and patients with no response were defined as “non-responders.”

### Measurement of serum IFX levels

Blood samples for measuring trough serum IFX levels were collected immediately before a new infusion. Serum IFX levels were measured using an LCMS-8060 quadrupole mass spectrometer (SHIMADZU, Kyoto, Japan), as previously reported, with some modifications [24–26]. Briefly, to obtain the peptides from the fragment antigen-binding (Fab) region of immunoglobulin G, serum samples were pretreated using the nSMOL^™^ Antibody BA Kit (SHIMADZU, Kyoto, Japan) according to the provided protocol. The lower limit of quantitation was 0.293 μg/mL.

### Detection of ADAs in serum

ADA analysis was performed by the electrochemiluminescence (ECL) method [27, 28]. A microplate coated with streptavidin (MSD GOLD 96-well Streptavidin QUICKPLEX Plate, Meso Scale Diagnostics [MSD], Rockville, MD, USA) was blocked with 150 μL blocking solution (3% MSD Blocker A) overnight at 4 °C. A master mixture of 20 μg/mL biotinylated IFX and 20 μg/mL ruthenium-labeled IFX was prepared in assay diluent (1% MSD Blocker A) at a ratio of 1:1. Subsequently, 25 μL of a diluted sample and 50 μL of the master mixture were added to each well in a 96-well plate, and incubated for 2 h under gentle agitation. After three washes with 200 μL of wash buffer (phosphate-buffered saline with 0.05% Tween 20), 50 μL of premix solution was transferred to each corresponding streptavidin-coated plate well, and the plates were incubated for 1 h under agitation. The plates were then washed three times, and 150 μL of read buffer (MSD Read Buffer T [4×] diluted two-fold in ultrapure water) was added to each well. The ECL signal from the solution was measured using a MESO QuickPlex SQ120 (MSD).

### Statistical analysis

Statistical analyses were performed using GraphPad Prism v7.0 (GraphPad Software, Inc., La Jolla, CA, USA). Non-normally distributed data were summarized with medians and analyzed using nonparametric tests (Mann-Whitney U test or Wilcoxon signed-rank test). Categorical data summarized with percentages were analyzed using Fisher’s exact test with continuity correction, where necessary. Results were considered statistically significant at *p*-value ≤0.05. The Kaplan-Meier method was performed to evaluate time to first response and time to loss of response.

To define an optimal cut-off value for predicting clinical response, a receiver operating characteristic (ROC) curve was plotted using JMP^®^ Pro14 (SAS Institute, Inc., Cary, NC, USA).

## Results

### Clinical efficacy and serum levels of IFX in RA patients

**Fig 2:**
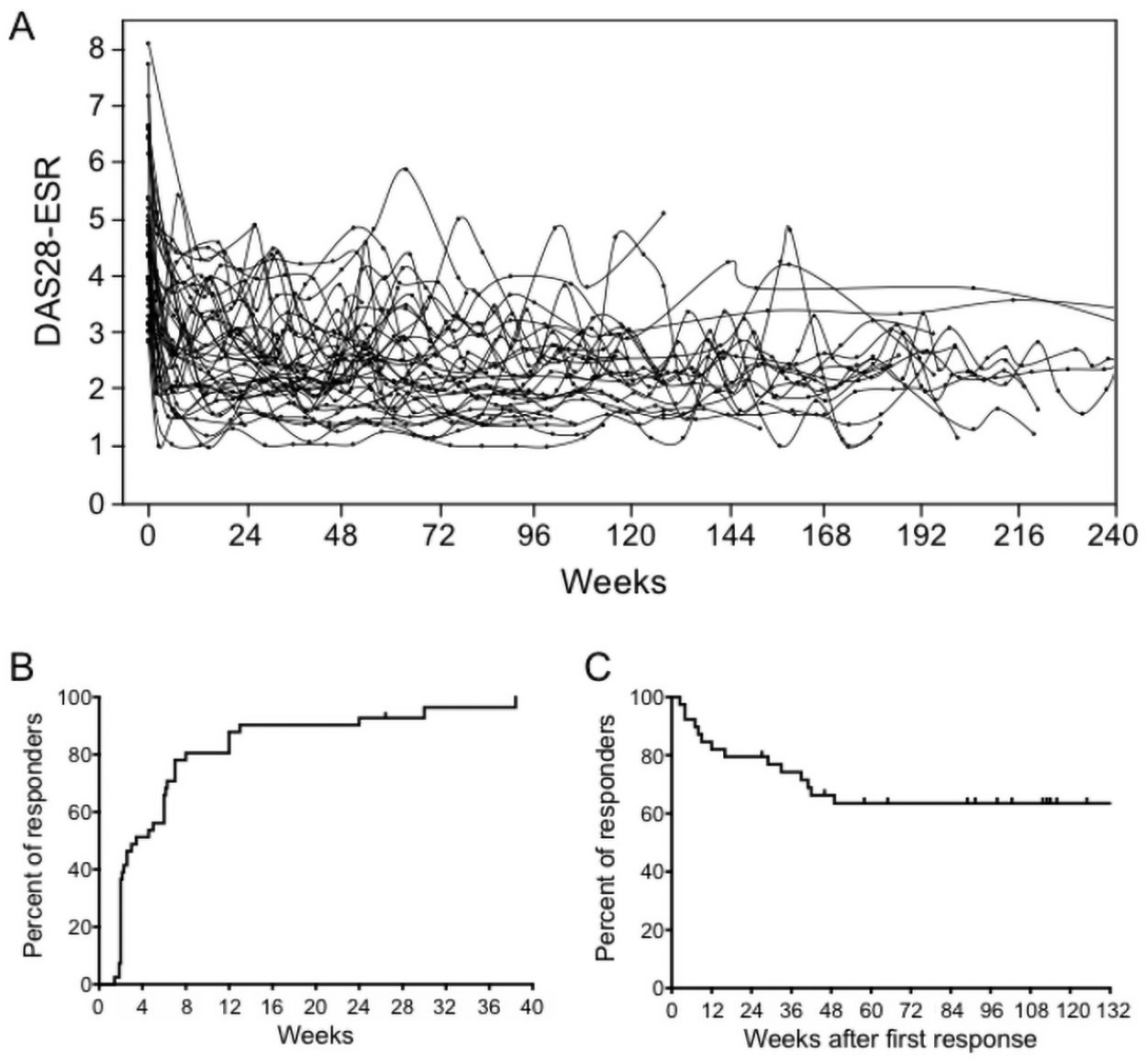
Clinical efficacy of IFX. (A) Change in DAS28-ESR over time. The x-axis represents time after introduction of IFX. The y-axis represents values of DAS28-ESR. (B) Kaplan-Meier curve showing time to first response. Responders represent patients with “good or moderate response” based on the EULAR response criteria. The baseline (Day 0) was defined as the day of IFX introduction. (C) Kaplan-Meier curve showing time to loss of response. A change from responder to non-responder was defined as “loss of response.” The baseline (Day 0) was defined as the time point when the first response was observed. Patients who never showed any response during observation periods were excluded (n = 40). Abbreviations: DAS28-ESR, the 28 joint disease activity score incorporating erythrocyte sedimentation rate; EULAR, European League Against Rheumatism; IFX, infliximab.

Fig 2A shows a change in DAS28-ESR after introduction of IFX. Large inter- and intra-individual differences in daily disease activities were observed. Kaplan-Meier curves indicated that more than 80% of total patients responded within 12 weeks after IFX introduction in clinical practice (Fig 2B), and around 40% of responders exhibited loss of response within 48 weeks after the first response (Fig 2C).

**Fig 3:**
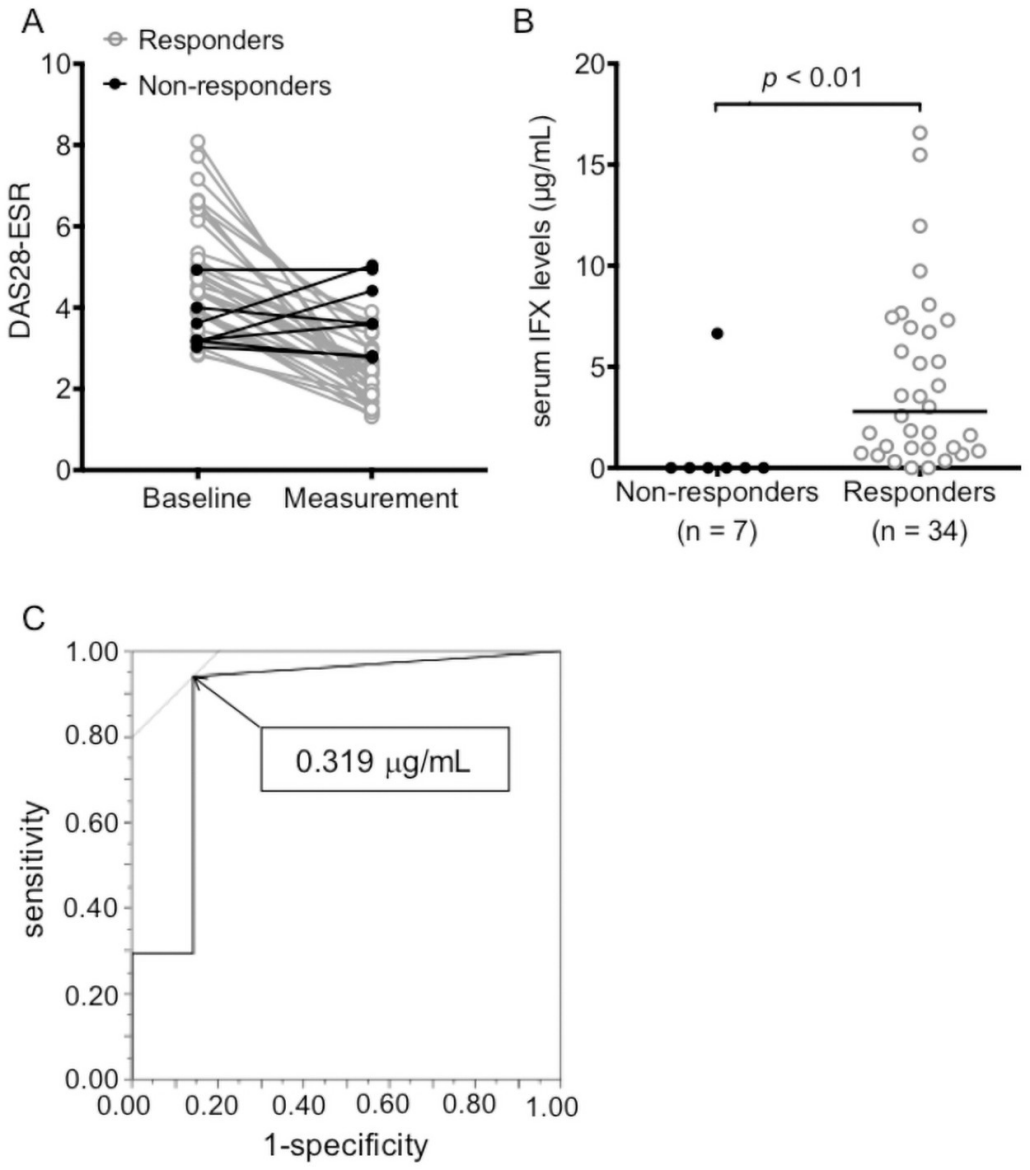
DAS28-ESR values and serum IFX concentrations in responders and non-responders. (A) DAS28-ESR values at baseline and at IFX measurement point. Responders (open circle) represent patients with “good or moderate response” and non-responders (closed circle) represent those with “no response” based on the European League Against Rheumatism (EULAR) response criteria. (B) Serum IFX levels were measured in non-responders and responders. Each dot represents each patient’s serum IFX level and the bars indicate the median. Differences between the groups were assessed by Mann-Whitney U test. (C) Receiver operating characteristic (ROC) curve for the determination of the optimal IFX cut-off value for predicting persistent responder (area under the curve (AUC) = 0.87). Abbreviations: DAS28-ESR, the 28 joint disease activity score incorporating erythrocyte sedimentation rate; IFX, infliximab.

There were 34 responders and 7 non-responders at the measurement point (Fig 3A). Serum IFX levels were significantly higher in responders than in non-responders (Fig 3B). The area under the curve (AUC) of the ROC curve was 0.87, and the cut-off value that distinguished EULAR responders from non-responders was 0.319 μg/mL (sensitivity: 94.1%, specificity: 85.7%, Fig 3C). Based on this, the cutoff value was set to 0.32 μg/mL for subsequent analyses.

### Background demographics and clinical characteristics of patients

Out of the 311 RA patients who received IFX therapy in the KURAMA cohort, 41 were eligible for the analysis. We assessed the risk of bias by comparing the baseline demographics and clinical characteristics of the patients between the total population and study cohort (S1 Table). There was a difference in year of IFX initiation, age, and swollen joint count (SJC). Patients were divided into two groups based on serum IFX levels, that is, patients with serum IFX level <0.32 μg/mL (Low-IFX group, n = 9) and patients with serum IFX level ≥0.32 μg/mL (High-IFX group, n = 32). The baseline demographics and clinical characteristics of the patients in the two groups are summarized in Table 1. At the measurement point, the mean duration of IFX treatment was around 1 year. Age, tender joint count, CDAI, SDAI, HAQ-DI and DAS28-ESR were significantly lower in the Low-IFX group than in the High-IFX group. Only patients in the High-IFX group used oral glucocorticoids, but there was no significant association between the use of glucocorticoids and disease severity. There were no significant differences in body weight, sex, disease duration, duration of IFX treatment, SJC, CRP level, RF-positive patients and concomitant MTX and csDMARDs use.

**Table 1:** Baseline demographics and clinical characteristics of the patients.

Characteristics	IFX ≤0.32 μg/mL (n = 9)	IFX >0.32 μg/mL (n = 32)	*p*-value
Age, mean (SD), (years)	47.4 (19.4)	61.6 (12.1)	0.03
Body weight, mean (SD), (kg)	57.8 (12.8)	55.8 (8.9)	0.85
Women, no. (%)	7 (77.8)	25 (78.1)	1.00
Disease duration, mean (SD), (years)	3.28 (2.01)	4.14 (3.71)	0.95
Duration of IFX treatment, median (Min-Max), (days)	332 (147–539)	429 (112–882)	0.34
Weekly MTX dose, mean (SD), (mg/week)	9.3 (3.0)	8.7 (3.5)	0.63
Oral glucocorticoid use, no. (%)	0 (0.0)	13 (40.6)	0.04
csDMARDs use, no. (%)	2 (22.2)	8 (25.0)	1.00
Tender joint count, mean (SD)	1.4 (1.2)	5.4 (5.8)	0.01
Swollen joint count, mean (SD)	1.9 (1.4)	5.4 (5.3)	0.07
CRP level, mean (SD), (mg/dL)	0.56 (0.61)	2.48 (3.42)	0.18
RF positive, no. (%)	8 (88.9)	22 (68.8)	0.24
CDAI, mean (SD)	9.9 (3.9)	20.4 (13.7)	<0.01
SDAI, mean (SD)	10.5 (4.1)	22.9 (15.9)	<0.01
HAQ-DI, mean (SD)	0.47 (0.35)	1.22 (0.99)	0.04
DAS28-ESR, mean (SD)	3.69 (0.65)	4.93 (1.43)	0.02

The patients were divided into two groups; Low-IFX (IFX ≤0.32 μg/mL) and High-IFX (IFX >0.32 μg/mL). Demographics and clinical characteristics at baseline are represented as means ± standard deviation (SD) for continuous data and numbers (percentages) for categorical data. Analysis of variance and Fisher’s exact test were used to compare the clinical characteristics among the different groups for continuous variables and categorical variables, respectively. csDMARDs include actarit, aurothiomalate, auranofin, bucillamine, iguratimod, leflunomide, mizoribine, salazosulfapyiridin, cyclosporine, and tacrolimus. Abbreviations: ACPA, anticyclic citrullinated peptide antibody; CDAI, clinical disease activity index; csDMARDs, conventional synthetic disease modifying anti-rheumatic drugs; CRP, C-reactive protein; DAS28-ESR, the 28 joint disease activity score incorporating erythrocyte sedimentation rate; HAQ, physical disability by health assessment questionnaire-disability index; IFX, infliximab; MTX, methotrexate; SDAI, simplified disease activity index; RF, rheumatoid factor.

### Disease activity markers in Low-IFX group and High-IFX group

**Fig 4:**
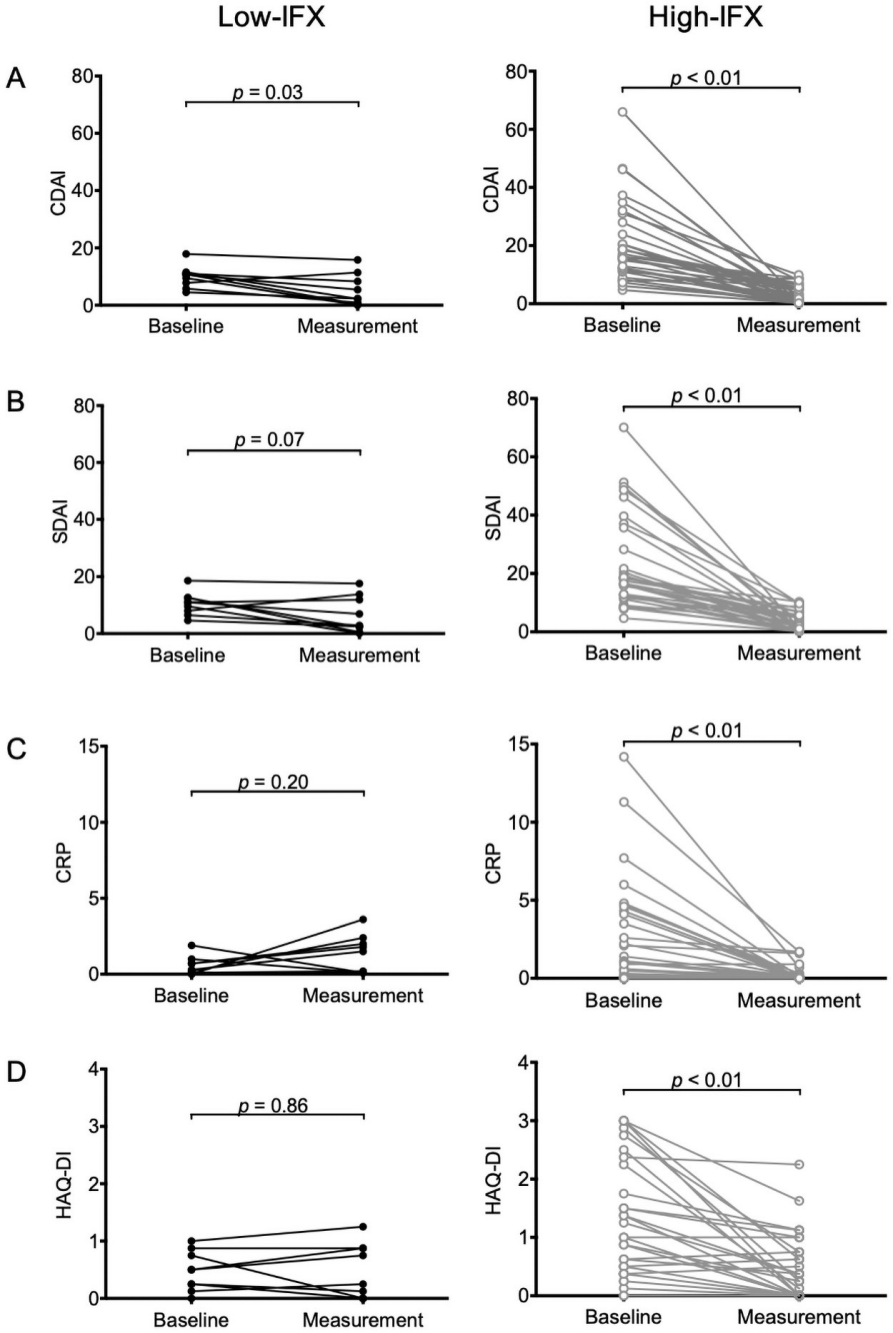
Changes in (A) CDAI, (B) SDAI, (C) CRP, and (D) HAQ-DI from the baseline to the IFX measurement point. The left figures show the data of patients with IFX level <0.32 μg/mL (closed circles), and the right figures show the data of patients with IFX level ≥0.32 μg/mL (open circles). Each line corresponds to each patient. The data were analyzed by Wilcoxon signed-rank test. Abbreviations: CDAI, clinical disease activity index; CRP, C-reactive protein; DAS28-ESR, the 28 joint disease activity score incorporating erythrocyte sedimentation rate; HAQ-DI, physical disability by health assessment questionnaire-disability index; IFX, infliximab; SDAI, simplified disease activity index.

In the present study, the “maximum effect point” was defined as the date when DAS28-ESR was the lowest during the IFX therapy between after its introduction point and at the measurement point. At the maximum effect point, only two patients (4.9%) were non-responders; there were no differences in proportions of responders between the Low-IFX and High-IFX groups (*p* = 0.40, Table 2). However, at the measurement point, five patients additionally turned into non-responder status, accordingly, there were significant differences in responder proportions between the Low-IFX group and the High-IFX group based on Fisher’s exact test (*p* <0.01). One non-responder in the High-IFX group had finally attained efficacy after the measurement point. Disease activity marker trends between the introduction and measurement points in the two groups are illustrated in Fig 4. CDAI and SDAI in the Low-IFX group improved significantly. In addition, CDAI, SDAI, CRP, and HAQ-DI scores in the High-IFX group exhibited notable improvements.

**Table 2:** Number (percentages) of responders and non-responders at maximum effect and measurement point in each group.

	Responders	Non-responders	*p*-value
**<maximum effect point>**			
IFX <0.32 μg/mL, n (%)	8 (88.9)	1 (11.1)	0.40
IFX ≥0.32 μg/mL, n (%)	31 (96.9)	1 (3.1)	
Total (%)	39 (95.1)	2 (4.9)	

**<measurement point>**			
IFX <0.32 μg/mL, n (%)	3 (33.3)	6 (66.7)	<0.01
IFX ≥0.32 μg/mL, n (%)	31 (96.9)	1 (3.1)	
Total (%)	34 (82.9)	7 (17.1)	

**<measurement point>**			
ADA-positive, n (%)	2 (50.0)	2 (50.0)	0.14
ADA-negative, n (%)	30 (85.7)	5 (14.3)	
Total (%)	32 (82.1)	7 (17.9)	

Responders had “good and moderate responses,” and non-responders had “no responses” based on the EULAR response criteria. ADAs in two patients could not be examined due to sample shortage. Values were considered statistically significant at a *p* value less than 0.05, based on two-sided Fisher’s exact test. Abbreviations: ADA, anti-drug antibody; EULAR, European League Against Rheumatism; IFX, infliximab.

### Correlation between serum IFX levels and ADA positivity

**Fig 5:**
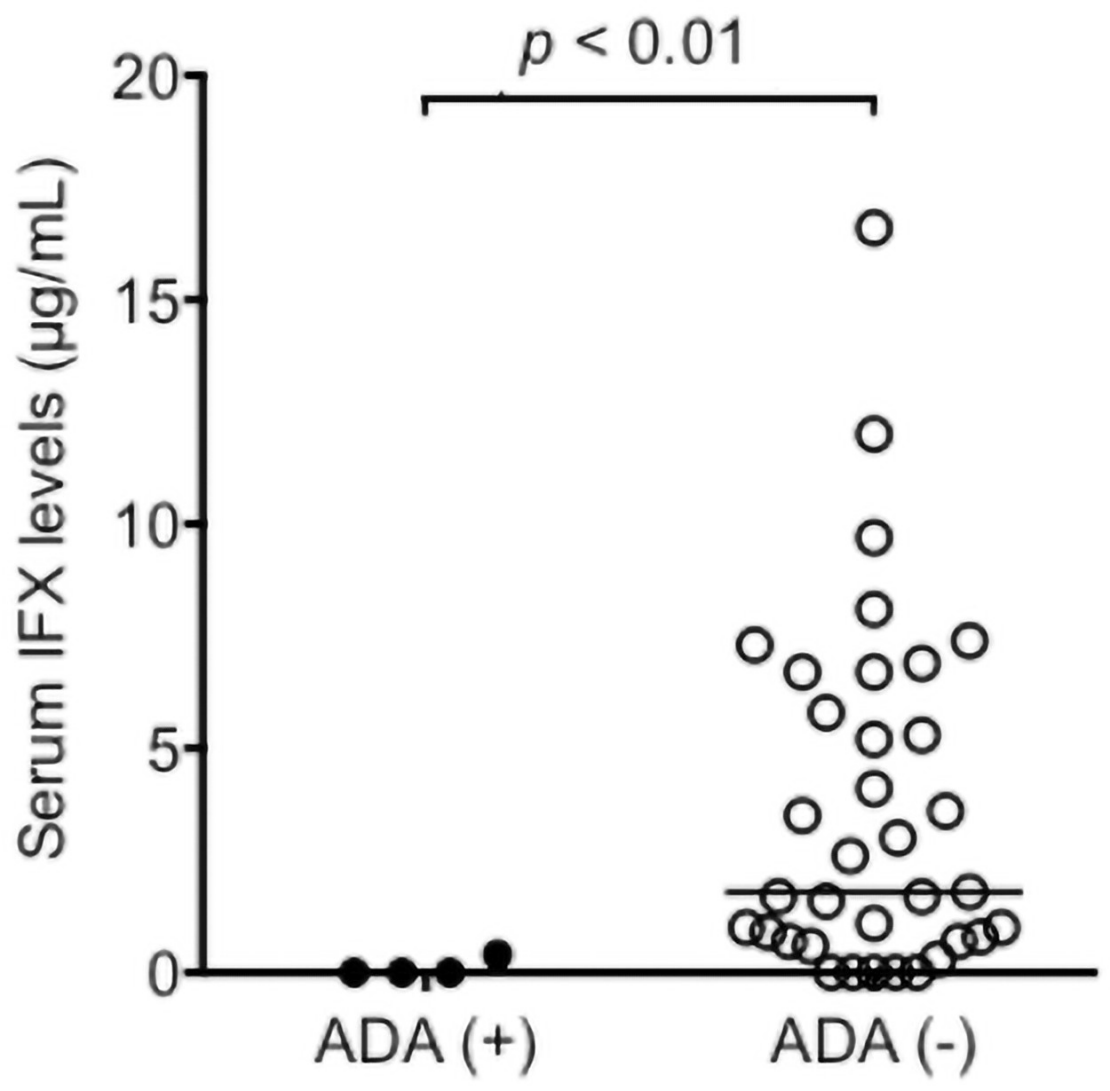
Comparison of serum IFX levels between ADA positive group (ADA (+)) and negative group (ADA (-)). Each dot represents each patient’s serum IFX level and the bars indicate the median. Differences between groups were assessed by Mann-Whitney U test. Abbreviations: ADA, anti-drug antibody; IFX, infliximab.

In 39 of the 41 investigated patients, serum samples were sufficient amount for the ADA determination. ADA was detected in four patients (10.3%) at the measurement point. All ADA-positive patients belonged to the steroid-free group. The IFX levels in the ADA-positive group were significantly lower than that in the ADA-negative group (*p* <0.01, Fig 5). Although two patients in the ADA-positive group (50.0%) were responders, 30 patients in the ADA-negative group (85.7%) were responders. There were no significant differences in proportions of responders between the ADA-positive group and ADA-negative group (Table 2).

## Discussion

In a previous intervention study (the RISING study), RA patients were randomly assigned to three treatment groups (3, 6, and 10 mg/kg IFX infusions) at week 10 after receiving 3 mg/kg IFX at weeks 0, 2, and 6 [20]. The rates of responders at week 54 for 3, 6 and 10 mg/kg were 10%, 56% and 100%, respectively. Better response was obtained in patients with higher dose of IFX. In addition, when the serum IFX concentration was ≥1.0 μg/mL, a clinical response was observed in 98.8% of patients. Although the exact therapeutic window of IFX is yet to be clearly defined, a higher trough level has been associated with improved clinical outcomes in several observational studies and post-hoc analyses of clinical trials for RA [21, 22] and other chronic inflammatory diseases [29–32]. Notably, our real-world cohort data indicated the effectiveness of IFX treatment in 39 of the 41 target patients (95.1%) at the point of maximum effect. The results obtained from this study strongly suggested that physicians increased IFX doses to appropriate levels in each patient even without measuring blood levels, and that primary ineffectiveness could be avoided in clinical practice.

Conversely, some patients showed secondary loss of response to IFX with the lapse of the continuous use. Notably, at the measurement point, the efficacy was significantly lower in the Low-IFX group than in the High-IFX group, strongly suggesting that that the effect of this therapy was potentially decreased by lower blood IFX level. It is challenging to reliably determine secondary ineffectiveness under long-term use. Information on serum IFX levels might be helpful for physicians in the assessment of patients. Overall, we propose the development of a treatment algorithm based on IFX TDM, wherein IFX therapeutic efficacy would be extensively re-evaluated when blood IFX concentrations are low under continuous use.

The determination of a cut-off value for predicting clinical response is a key challenge. In the RISING study, a trough serum IFX level of 1.0 μg/mL was the threshold level for eliciting clinical responses [20]. Wolbink *et al*. [33] reported similar results, where patients with low trough serum IFX levels (less than 1.2 μg/mL) showed relatively low improvements in DAS28 score. From the result of ROC analysis in this study, an optimal cut-off value of ≥0.32 μg/mL was determined. Our study also revealed almost similar results when the cut-off value was determined to be at serum IFX level ≥1.0 μg/mL (S2 Table), which is largely consistent with RISING study [20]. Enzyme-linked immunosorbent assay method has been extensively used to quantify serum therapeutic antibodies. However, by use of this technique, nonspecific signals could be detected [34, 35]. In the present study, we employed a liquid chromatography-tandem mass spectrometry (LC-MS/MS) method with nano-surface and molecular-orientation limited proteolysis to monitor IFX-specific peptides, based on Food and Drug Administration (FDA) criteria [36]. The analytical methods used should be taken into account to set cut-off values in clinical practice. Further studies are required to determine the optimal cut-off values across several analytical methods.

Previous studies have shown that ADA is one of the factors influencing IFX pharmacokinetics [9–11]. ADA formation increases IFX clearance, which can, in turn, reduce serum IFX levels. In the present study, 4 out of 39 patients (10.3%) were ADA-positive. Compared to the ADA-negative patients, the ADA-positive patients had significantly lower serum IFX levels. All ADA-positive patients belonged to the steroid-free group, which may have caused the significant difference in glucocorticoid use between the High- and Low-IFX groups. The proportion of patients satisfying the EULAR response criteria tended to be lower in the ADA-positive group. Although ADA could influence IFX pharmacokinetics, the key factor influencing IFX efficacy is serum IFX level. Although there are factors other than ADA to influence blood IFX levels, monitoring IFX levels is the potentially optimal tool for evaluating its clinical efficacy. Conversely, it has been reported that dose escalation of IFX in patients with IBD could be less successful for improving treatment efficacy in ADA-positive patients compared to that in ADA-negative patients [37, 38]. Measurement of ADA as well as serum IFX concentrations, could facilitate determination of the next appropriate therapeutic strategy between dose escalation or switching therapies in patients exhibiting secondary loss of response.

The present study had some limitations. First, the sample size was small. We had to exclude numerous patients with no information on serum IFX level or DAS28-ESR data around the IFX administration date as clinical data prior to 2011 was not included in the KURAMA cohort. Baseline demographics and clinical characteristics of the patients between the total population and study cohort were almost similar; however, selection bias cannot be completely precluded. Second, we did not measure serum IFX levels at the maximum effect point and were unable to investigate the association between reduction in DAS28-ESR and serum IFX levels at the maximum effect point. Third, background characteristics in several patients were different between the High-IFX and Low-IFX groups. The present study was an observational study, and we could not employ randomization to control or eliminate confounding factors. However, more than 90% of patients in both the High- and Low-IFX groups exhibited a primary response. The skewed patient characteristic distributions could have had relatively less impact on our results associated with secondary non-response.

## Conclusions

The present study demonstrated that serum IFX levels were correlated with IFX therapeutic efficacy under continuous use, based on real-world cohort data. In clinical practice, the IFX primary ineffectiveness could be avoided via appropriate dose escalation without measuring the blood concentrations. However, IFX TDM could facilitate the identification of secondary non-response and, in turn, proper IFX use.

## Supporting information

S1 Table Baseline demographics and clinical characteristics of the patients in total and study cohort. Demographics and clinical characteristics at baseline are represented as means ± standard deviation (SD) for continuous data and numbers (percentages) for categorical data. Analysis of variance and Chi-square test were used to compare the clinical characteristics among the different groups for continuous variables and categorical variables, respectively. csDMARDs include actarit, aurothiomalate, auranofin, bucillamine, iguratimod, leflunomide, mizoribine, salazosulfapyiridin, cyclosporine, and tacrolimus. Abbreviations: CDAI, clinical disease activity index; csDMARDs, conventional synthetic disease modifying anti-rheumatic drugs; CRP, C-reactive protein; DAS28-ESR, the 28 joint disease activity score incorporating erythrocyte sedimentation rate; HAQ-DI, physical disability by health assessment questionnaire-disability index; IFX, infliximab; MTX, methotrexate; SDAI, simplified disease activity index; RF, rheumatoid factor.
(DOCX)

S2 Table Number of responders and non-responders at maximum effect and measurement points in High/Low-IFX group. The cut-off value was determined to be at serum IFX level ≥1.0 μg/mL. Responders had “good and moderate responses” and non-responders had “no responses” based on the EULAR response criteria. Values were considered statistically significant at a *p* value less than 0.05, based on two-sided Fisher’s exact test. Abbreviations: EULAR, European League Against Rheumatism; IFX, infliximab.
(DOCX)

## References

[1] Maini R, St Clair EW, Breedveld F, Furst D, Kalden J, Weisman M, et al. Infliximab (chimeric anti-tumour necrosis factor alpha monoclonal antibody) versus placebo in rheumatoid arthritis patients receiving concomitant methotrexate: a randomised phase III trial. ATTRACT Study Group. Lancet. 1999;354(9194): 1932–9. doi: 10.1016/s0140-6736(99)05246-0 10622295

[2] Lipsky PE, van der Heijde DM, St Clair EW, Furst DE, Breedveld FC, Kalden JR, et al. Infliximab and methotrexate in the treatment of rheumatoid arthritis. Anti-Tumor Necrosis Factor Trial in Rheumatoid Arthritis with Concomitant Therapy Study Group. N Engl J Med. 2000;343(22): 1594–602. 11096166 10.1056/NEJM200011303432202

[3] Hanauer S, Wagner C, Bala M, Mayer L, Travers S, Diamond R, et al. Incidence and importance of antibody responses to infliximab after maintenance or episodic treatment in Crohn’s disease. Clin Gastroenterol Hepatol. 2004;2(7): 542–53. doi: 10.1016/s1542-3565(04)00238-1 15224278

[4] Chaudhari U, Romano P, Mulcahy L, Dooley L, Baker D, Gottlieb A. Efficacy and safety of infliximab monotherapy for plaque-type psoriasis: a randomised trial. Lancet. 2001;357(9271): 1842–7. doi: 10.1016/s0140-6736(00)04954-0 11410193

[5] Rutgeerts P, Sandborn W, Feagan B, Reinisch W, Olson A, Johanns J, et al. Infliximab for Induction and Maintenance Therapy for Ulcerative Colitis. N Engl J Med. 2005;353(23): 2462–76. doi: 10.1056/NEJMoa050516 16339095

[6] Yanai H, Hanauer S. Assessing response and loss of response to biological therapies in IBD. Am J Gastroenterol. 2011;106(4): 685–98. doi: 10.1038/ajg.2011.103 21427713

[7] Buch M, Bingham S, Bryer D, Emery P. Long-term infliximab treatment in rheumatoid arthritis: subsequent outcome of initial responders. Rheumatology (Oxford). 2007;46(7): 1153–6. doi: 10.1093/rheumatology/kem075 17478470

[8] Ebina K, Hashimoto M, Yamamoto W, Ohnishi A, Kabata D, Hirano T, et al. Drug Retention and Discontinuation Reasons Between Seven Biologics in Patients With Rheumatoid Arthritis -The ANSWER Cohort Study. PLoS One. 2018;13(3). Epub e0194130. doi: 10.1371/journal.pone.0194130 29543846 PMC5854351

[9] Baert F, Noman M, Vermeire S, Van Assche G, D’ Haens G, Carbonez A, et al. Influence of Immunogenicity on the Long-Term Efficacy of Infliximab in Crohn’s Disease. N Engl J Med. 2003;348(7): 601–8. doi: 10.1056/NEJMoa020888 12584368

[10] Plasencia C, Jurado T, Villalba A, Peitedado D, Casla MT, Nuno L, et al. Effect of Infliximab Dose Increase in Rheumatoid Arthritis at Different Trough Concentrations: A Cohort Study in Clinical Practice Conditions. Front Med. 2015;2: 71. doi: 10.3389/fmed.2015.00071 26501060 PMC4597116

[11] Kalden JR, Schulze-Koops H. Immunogenicity and loss of response to TNF inhibitors: implications for rheumatoid arthritis treatment. Nat Rev Rheumatol. 2017;13(12): 707–18. doi: 10.1038/nrrheum.2017.187 29158574

[12] Bendtzen K, Geborek P, Svenson M, Larsson L, Kapetanovic MC, Saxne T. Individualized monitoring of drug bioavailability and immunogenicity in rheumatoid arthritis patients treated with the tumor necrosis factor alpha inhibitor infliximab. Arthritis Rheum. 2006;54(12): 3782–9. doi: 10.1002/art.22214 17133559

[13] Pascual-Salcedo D, Plasencia C, Ramiro S, Nuno L, Bonilla G, Nagore D, et al. Influence of immunogenicity on the efficacy of long-term treatment with infliximab in rheumatoid arthritis. Rheumatology (Oxford). 2011;50(8): 1445–52. doi: 10.1093/rheumatology/ker124 21427177

[14] Strand V, Balsa A, Al-Saleh J, Barile-Fabris L, Horiuchi T, Takeuchi T, et al. Immunogenicity of Biologics in Chronic Inflammatory Diseases: A Systematic Review. BioDrugs. 2017;31(4): 299–316. doi: 10.1007/s40259-017-0231-8 28612180 PMC5548814

[15] Siljehult F, Arlestig L, Eriksson C, Rantapaa-Dahlqvist S. Concentrations of infliximab and anti-drug antibodies in relation to clinical response in patients with rheumatoid arthritis. Scand J Rheumatol. 2018;47(5):345–50. doi: 10.1080/03009742.2018.1433232 29701536

[16] Takeuchi T, Miyasaka N, Tatsuki Y, Yano T, Yoshinari T, Abe T, et al. Baseline tumour necrosis factor alpha levels predict the necessity for dose escalation of infliximab therapy in patients with rheumatoid arthritis. Ann Rheum Dis. 2011;70(7): 1208–15. doi: 10.1136/ard.2011.153023 21478189 PMC3103666

[17] Tzaban S, Massol R, Yen E, Hamman W, Frank S, Lapierre L, et al. The recycling and transcytotic pathways for IgG transport by FcRn are distinct and display an inherent polarity. J Cell Bio. 2009;185(4): 673–84. doi: 10.1083/jcb.200809122 19451275 PMC2711563

[18] Kuo T, Aveson V. Neonatal Fc receptor and IgG-based therapeutics. MAbs. 2011;3(5): 422–30. doi: 10.4161/mabs.3.5.16983 22048693 PMC3225846

[19] Gill KL, Machavaram KK, Rose RH, Chetty M. Potential Sources of Inter-Subject Variability in Monoclonal Antibody Pharmacokinetics. Clin Pharmacokinet. 2016;55(7): 789–805. doi: 10.1007/s40262-015-0361-4 26818483

[20] Takeuchi T, Miyasaka N, Inoue K, Abe T, Koike T, study R. Impact of trough serum level on radiographic and clinical response to infliximab plus methotrexate in patients with rheumatoid arthritis: results from the RISING study. Mod Rheumatol. 2009;19(5): 478–87. doi: 10.1007/s10165-009-0195-8 19626391 PMC2759008

[21] Mulleman D, Meric JC, Paintaud G, Ducourau E, Magdelaine-Beuzelin C, Valat JP, et al. Infliximab concentration monitoring improves the control of disease activity in rheumatoid arthritis. Arthritis Res Ther. 2009;11(6): R178. doi: 10.1186/ar2867 19939280 PMC3003525

[22] Price S. Rheumatoid arthritis: monitoring serum concentration of infliximab might improve RA disease control. Nat Rev Rheumatol. 2010;6(2): 66. Epub 2010/10/27. doi: 10.1038/nrrheum.2009.271 20976866

[23] Terao C, Hashimoto M, Yamamoto K, Murakami K, Ohmura K, Nakashima R, et al. Three groups in the 28 joints for rheumatoid arthritis synovitis—analysis using more than 17,000 assessments in the KURAMA database. PLoS One. 2013;8(3). doi: 10.1371/journal.pone.0059341 23555018 PMC3595245

[24] Iwamoto N, Shimada T, Umino Y, Aoki C, Aoki Y, Sato TA, et al. Selective detection of complementarity-determining regions of monoclonal antibody by limiting protease access to the substrate: nano-surface and molecular-orientation limited proteolysis. Analyst. 2014;139(3): 576–80. Epub 2013/12/12. doi: 10.1039/c3an02104a 24326404

[25] Iwamoto N, Yokoyama K, Takanashi M, Yonezawa A, Matsubara K, Shimada T. Verification between Original and Biosimilar Therapeutic Antibody Infliximab Using nSMOL Coupled LC-MS Bioanalysis in Human Serum. Curr Pharm Biotechnol. 2018;19(6): 495–505. Epub 2018/07/04. doi: 10.2174/1389201019666180703093517 29968534 PMC6198460

[26] Iwamoto N, Takanashi M, Yokoyama K, Yonezawa A, Denda M, Hashimoto M, et al. Multiplexed monitoring of therapeutic antibodies for inflammatory diseases using Fab-selective proteolysis nSMOL coupled with LC-MS. J Immunol Methods. 2019;472: 44–54. doi: 10.1016/j.jim.2019.06.014 31201793

[27] Reinisch W, Jahnsen J, Schreiber S, Danese S, Panés J, Balsa A, et al. Evaluation of the Cross-reactivity of Antidrug Antibodies to CT-P13 and Infliximab Reference Product (Remicade): An Analysis Using Immunoassays Tagged with Both Agents. BioDrugs. 2017;31(3): 223–37. doi: 10.1007/s40259-017-0219-4 28497221 PMC5443869

[28] Shibata H, Nishimura K, Miyama C, Tada M, Suzuki T, Saito Y, et al. Comparison of different immunoassay methods to detect human anti-drug antibody using the WHO erythropoietin antibody reference panel for analytes. J Immunol Methods. 2018;452: 73–7. doi: 10.1016/j.jim.2017.09.009 28970009

[29] Pallagi-Kunstar E, Farkas K, Szepes Z, Nagy F, Szucs M, Kui R, et al. Utility of serum TNF-alpha, infliximab trough level, and antibody titers in inflammatory bowel disease. World J Gastroenterol. 2014;20(17): 5031–5. Epub 2014/05/17. doi: 10.3748/wjg.v20.i17.5031 24833846 PMC4009537

[30] Dannepond C, Maruani A, Machet L, Ternant D, Paintaud G, Samimi M. Serum infliximab concentrations in psoriatic patients treated with infliximab: a systematic review. Acta Derm Venereol. 2015;95(4). doi: 10.2340/00015555-1980 25270995

[31] Vande Casteele N, Ferrante M, Van Assche G, Ballet V, Compernolle G, Van Steen K, et al. Trough concentrations of infliximab guide dosing for patients with inflammatory bowel disease. Gastroenterology. 2015;148(7): 1320–9.e3. Epub 2015/03/01. doi: 10.1053/j.gastro.2015.02.031 25724455

[32] Warman A, Straathof J, Derijks L. Therapeutic drug monitoring of infliximab in inflammatory bowel disease patients in a teaching hospital setting: results of a prospective cohort study. Eur J Gastroenterol Hepatol. 2015;27(3): 242–8. doi: 10.1097/MEG.0000000000000279 25569569

[33] Wolbink GJ, Voskuyl AE, Lems WF, de Groot E, Nurmohamed MT, Tak PP, et al. Relationship between serum trough infliximab levels, pretreatment C reactive protein levels, and clinical response to infliximab treatment in patients with rheumatoid arthritis. Ann Rheum Dis. 2005;64(5): 704–7. Epub 2004/10/16. doi: 10.1136/ard.2004.030452 15485995 PMC1755482

[34] Güven E, Duus K, Lydolph M, Jørgensen C, Laursen I, Houen G. Non-specific binding in solid phase immunoassays for autoantibodies correlates with inflammation markers. J Immunol Methods. 2014;403(1–2): 26–36. doi: 10.1016/j.jim.2013.11.014 24287423

[35] Jourdil J, Lebert D, Gautier-Veyret E, Lemaitre F, Bonaz B, Picard G, et al. Infliximab quantitation in human plasma by liquid chromatography-tandem mass spectrometry: towards a standardization of the methods? Anal Bioanal Chem. 2017;409(5): 1195–205. doi: 10.1007/s00216-016-0045-4 27826630

[36] U.S. Food and Drug Administration. Bioanalytical Method Validation Guidance for Industry. 2018 [Internet. Accessed January 5, 2021.] https://www.fda.gov/regulatory-information/search-fda-guidance-documents/bioanalytical-method-validation-guidance-industry.

[37] Kothari M, Nguyen D, Parekh N. Strategies for overcoming anti-tumor necrosis factor drug antibodies in inflammatory bowel disease: Case series and review of literature. World J Gastrointest Pharmacol Ther. 2017;8(3): 155–61. doi: 10.4292/wjgpt.v8.i3.155 28828193 PMC5547373

[38] Afif W, Loftus E, Faubion W, Kane S, Bruining D, Hanson K, et al. Clinical Utility of Measuring Infliximab and Human Anti-Chimeric Antibody Concentrations in Patients With Inflammatory Bowel Disease. Am J Gastroenterol. 2010;105(5): 1133–9. doi: 10.1038/ajg.2010.9 20145610 PMC6937708

