# Supplementary material for: Potential application of measuring serum infliximab levels in rheumatoid arthritis management: A retrospective study based on KURAMA cohort data
Source: PLoS One. 2021 Oct 13;16(10):e0258601. doi: 10.1371/journal.pone.0258601 (PMC8513849; doi:10.1371/journal.pone.0258601)
Supplement: S1 Table — Demographics and clinical characteristics at baseline are represented as means ± standard deviation (SD) for continuous data and numbers (percentages) for categorical data. Analysis of variance and Chi-square test were used to compare the clinical characteristics among the different groups for continuous variables and categorical variables, respectively. csDMARDs include actarit, aurothiomalate, auranofin, bucillamine, iguratimod, leflunomide, mizoribine, salazosulfapyiridin, cyclosporine, and tacrolimus. Abbreviations: CDAI, clinical disease activity index; csDMARDs, conventional synthetic disease modifying anti-rheumatic drugs; CRP, C-reactive protein; DAS28-ESR, the 28 joint disease activity score incorporating erythrocyte sedimentation rate; HAQ-DI, physical disability by health assessment questionnaire-disability index; IFX, infliximab; MTX, methotrexate; SDAI, simplified disease activity index; RF, rheumatoid factor. (DOCX) [file pone.0258601.s001.docx]

**S1 Table. Baseline demographics and clinical characteristics of the patients in total population and study cohort.**

| Characteristics | Total population (n = 311) | | Study cohort (n = 41) | | *p*-value |
| --- | --- | --- | --- | --- | --- |
| Year of IFX initiation | n = 299 |  | n = 41 |  | *< 0.01* |
| – 2010/12/31, no. (%) |  | 157 (52.5) |  | 0 (0.0) |  |
| 2011/1/1 – 2014/12/31, no. (%) |  | 105 (35.1) |  | 26 (63.4) |  |
| 2015/1/1 – 2018/12/31, no. (%) |  | 37 (12.4) |  | 15 (36.6) |  |
| Age, mean (SD), (years) | n = 299 | 53.4 (14.5) | n = 41 | 58.5 (14.9) | *0.016* |
| Body weight, mean (SD), (kg) | n = 210 | 56.4 (10.7) | n = 41 | 56.2 (9.7) | 0.96 |
| Female, no. (%) | n = 311 | 242 (77.8) | n = 41 | 32 (78.0) | 0.97 |
| Disease duration, mean (SD), (years) | n = 271 | 7.1 (8.7) | n = 41 | 3.9 (3.4) | 0.16 |
| Duration of IFX treatment at measurement point, median (Min-Max), (days) | ND | ND | n = 41 | 385 (112-882) | ND |
| Weekly MTX dose, mean (SD), (mg/week) | n = 136 | 7.8 (4.3) | n = 41 | 8.8 (3.4) | 0.26 |
| Oral glucocorticoid use, no. (%) | n = 136 | 39 (28.7) | n = 41 | 13 (31.7) | 0.71 |
| csDMARDs use, no. (%) | n = 136 | 28 (20.6) | n = 41 | 10 (24.4) | 0.60 |
| Tender joint count, mean (SD) | n = 104 | 3.7 (5.0) | n = 41 | 4.5 (5.4) | 0.19 |
| Swollen joint count, mean (SD) | n = 104 | 3.5 (4.7) | n = 41 | 4.6 (4.9) | *0.039* |
| CRP level, mean (SD), (mg/dL) | n = 104 | 1.73 (2.82) | n = 41 | 2.06 (3.13) | 0.52 |
| RF positive, no. (%) | n = 104 | 72 (69.2) | n = 41 | 30 (73.2) | 0.64 |
| CDAI, mean (SD) | n = 103 | 15.4 (12.6) | n = 41 | 18.1 (13.0) | 0.12 |
| SDAI, mean (SD) | n = 103 | 17.1 (14.3) | n = 41 | 20.2 (15.1) | 0.11 |
| HAQ-DI, mean (SD) | n = 104 | 0.93 (0.90) | n = 41 | 1.06 (0.94) | 0.39 |
| DAS28-ESR, mean (SD) | n = 103 | 4.10 (1.63) | n = 41 | 4.66 (1.39) | 0.067 |

Demographics and clinical characteristics at baseline are represented as means ± standard deviation (SD) for continuous data and numbers (percentages) for categorical data. Analysis of variance and Chi-square test were used to compare the clinical characteristics among the different groups for continuous variables and categorical variables, respectively. csDMARDs include actarit, aurothiomalate, auranofin, bucillamine, iguratimod, leflunomide, mizoribine, salazosulfapyiridin, cyclosporine, and tacrolimus. Abbreviations: CDAI, clinical disease activity index; csDMARDs, conventional synthetic disease modifying anti-rheumatic drugs; CRP, C-reactive protein; DAS28-ESR, the 28 joint disease activity score incorporating erythrocyte sedimentation rate; HAQ-DI, physical disability by health assessment questionnaire-disability index; IFX, infliximab; MTX, methotrexate; SDAI, simplified disease activity index; RF, rheumatoid factor.
