# Supplementary material for: Potential application of measuring serum infliximab levels in rheumatoid arthritis management: A retrospective study based on KURAMA cohort data
Source: PLoS One. 2021 Oct 13;16(10):e0258601. doi: 10.1371/journal.pone.0258601 (PMC8513849; doi:10.1371/journal.pone.0258601)
Supplement: S2 Table — The cut-off value was determined to be at serum IFX level ≥1.0 μg/mL. Responders had “good and moderate responses” and non-responders had “no responses” based on the EULAR response criteria. Values were considered statistically significant at a p value less than 0.05, based on two-sided Fisher’s exact test. Abbreviations: EULAR, European League Against Rheumatism; IFX, infliximab. (DOCX) [file pone.0258601.s002.docx]

**S2 Table. Number of responders and non-responders at maximum effect and measurement points in High/Low-IFX group.**

|  | Responders | Non-responders | *p*-value |
| --- | --- | --- | --- |
| **<maximum effect point>** | | | |
| IFX < 1.0 µg/mL, n (%) | 15 (93.8) | 1 (6.2) | > 0.99 |
| IFX ≥ 1.0 µg/mL, n (%) | 24 (96.0) | 1 (4.0) |  |
| Total (%) | 39 (95.1) | 2 (4.9) |  |
| **<measurement point>** | | | |
| IFX < 1.0 µg/mL, n (%) | 10 (62.5) | 6 (37.5) | < 0.01 |
| IFX ≥ 1.0 µg/mL, n (%) | 24 (96.0) | 1 (4.0) |  |
| Total (%) | 34 (82.9) | 7 (17.1) |  |

The cut-off value was determined to be at serum IFX level ≥ 1.0 µg/mL. Responders had “good and moderate responses” and non-responders had “no responses” based on the EULAR response criteria. Values were considered statistically significant at a *p* value less than 0.05, based on two-sided Fisher’s exact test. Abbreviations: EULAR, European League Against Rheumatism; IFX, infliximab.
